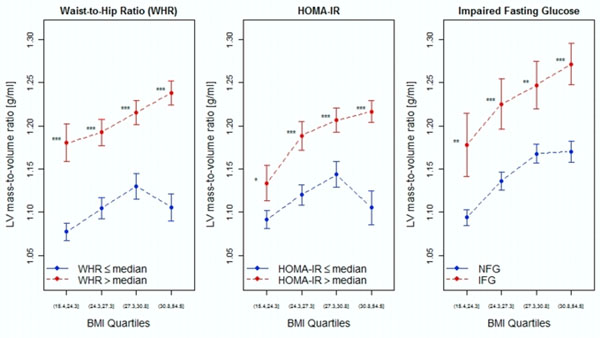# Insulin resistance, subclinical left ventricular remodeling, and the obesity paradox: the multi-ethnic study of atherosclerosis

**DOI:** 10.1186/1532-429X-15-S1-O20

**Published:** 2013-01-30

**Authors:** Ravi Shah, Siddique Abbasi, Bobby Heydari, Carsten Rickers, David R Jacobs, Lu Wang, Raymond Y Kwong, David A Bluemke, Joao A Lima, Michael Jerosch-Herold

**Affiliations:** 1 Brigham and Women's Hospital, Boston, MA, USA; 2 Department of Pediatric Cardiology, University Hospital of Schleswig-Holstein, Campus Kiel, Kiel, Germany; 3 University of Minnesota, School of Public Health, Division of Epidemiology and Community Health, Minneapolis, MN, USA; 4 Harvard School of Public Health, Department of Epidemiology and Biostatistics, Boston, MA, USA; 5 Radiology and Imaging Sciences, National Institutes of Health Clinical Center, National Institute of Biomedical Imaging and Bioengineering, Bethesda, MD, USA

## Background

Recent studies suggest that central obesity and insulin resistance may be primary mediators of obesity-related cardiac remodeling independent of body mass index (BMI). We assessed in the Multi-Ethnic Study of Atherosclerosis (MESA) whether insulin resistance and waist-to-hip ratio had effects on cardiac remodeling, independent of obesity.

## Methods

We investigated 4,364 individuals without diabetes in MESA. Insulin resistance (by impaired fasting glucose, IFG: 100-125 mg/dl or homeostatic model assessment of insulin resistance, HOMA-IR) and waist-to-hip ratio (WHR) were used for cardiometabolic phenotyping. Multivariate linear regression analysis was used to determine the effects of the cardiometabolic markers on LV remodeling, assessed primarily through the LV mass-to-volume ratio obtained by cine cardiac magnetic resonance imaging.

## Results

Individuals with IFG were more likely to be older, hypertensive, with increased prevalence of cardiometabolic risk factors regardless of BMI. In each quartile of BMI, individuals with above-median HOMA-IR, above-median WHR, or IFG had a higher LV mass-to-volume ratio (p<0.05 for all). HOMA-IR (p<0.0001), WHR (p<0.0001), and the presence of IFG (p=0.04), but not BMI (p=0.24), were independently associated with LV mass-to-volume ratio after adjustment for age, gender, hypertension, race, and dyslipidemia.

## Conclusions

Insulin resistance and waist-to-hip ratio are associated with concentric LV remodeling independent of BMI. These results support the emerging hypothesis that the cardiometabolic phenotype, defined by insulin resistance and central obesity, may play a critical role in LV remodeling independently of BMI.

## Funding

MESA was supported by contracts NO1-HC-95159 through N01-HC-95169 from the National Heart, Lung, and Blood Institute. Dr. Shah is supported by an American Heart Association Post-Doctoral Fellowship Award (11POST000002) and a training grant from the Heart Failure National Institutes of Health Clinical Research Network (U01-HL084877). Dr. Jerosch-Herold receives support through R01-HL-65580. All other authors have no financial disclosures relevant to the content of this manuscript.

**Figure 1:**